# A Discrete Dipole Approximation Solver Based on the COCG-FFT Algorithm and Its Application to Microwave Breast Imaging

**DOI:** 10.1155/2019/9014969

**Published:** 2019-07-17

**Authors:** Samar Hosseinzadegan, Andreas Fhager, Mikael Persson, Paul Meaney

**Affiliations:** 1 Electrical Engineering Department, Chalmers University of Technology, 41296 Gothenburg, Sweden; 2 The Thayer School of Engineering, Dartmouth College, Hanover, NH 03755, USA

## Abstract

We introduce the discrete dipole approximation (DDA) for efficiently calculating the two-dimensional electric field distribution for our microwave tomographic breast imaging system. For iterative inverse problems such as microwave tomography, the forward field computation is the time limiting step. In this paper, the two-dimensional algorithm is derived and formulated such that the iterative conjugate orthogonal conjugate gradient (COCG) method can be used for efficiently solving the forward problem. We have also optimized the matrix-vector multiplication step by formulating the problem such that the nondiagonal portion of the matrix used to compute the dipole moments is block-Toeplitz. The computation costs for multiplying the block matrices times a vector can be dramatically accelerated by expanding each Toeplitz matrix to a circulant matrix for which the convolution theorem is applied for fast computation utilizing the fast Fourier transform (FFT). The results demonstrate that this formulation is accurate and efficient. In this work, the computation times for the direct solvers, the iterative solver (COCG), and the iterative solver using the fast Fourier transform (COCG-FFT) are compared with the best performance achieved using the iterative solver (COCG-FFT) in C++. Utilizing this formulation provides a computationally efficient building block for developing a low cost and fast breast imaging system to serve under-resourced populations.

## 1. Introduction

The mortality rate due to the breast cancer in women worldwide has led numerous research groups to investigate early diagnosis programs. Along with other imaging methods such as X-ray computed tomography [1], positron emission tomography [2], and magnetic resonance imaging [3, 4], microwave imaging has been tested in multiple settings. In this context, microwave imaging is performed in four primary forms: radar, holography, thermoacoustic imaging, and tomography. The radar approaches have been studied in an array of simulation experiments and have advanced to several clinical tests [5–8]. Holography approaches have been primarily tested in simulation and phantom experiments [9, 10]. Thermoacoustic imaging work has been evaluated in several phantom experiments along with early clinical studies [11, 12]. In this report, we focus on microwave tomography (MWT) which has been tested in several breast imaging clinical trials and has provided relevant diagnostic information regarding diagnosis of cancer and monitoring of tumor progression during neoadjuvant chemotherapy [13, 14].

In spite of the demand and interest for microwave tomography, the computational costs of various algorithms have remained a primary obstacle in translation to real applications [15, 16]. The choice of three or two-dimensional imaging algorithms has considerable impact on the computation time and necessary hardware resources [17, 18]. Investigations into 3D imaging are considerably more common than that for 2D because of the superior measurement model match despite the associated costs [16, 19]. For many groups investigating microwave imaging, the amount of measurement data required is often a significant barrier to real 3D implementations [20, 21]. For most numerical techniques, solving the 3D imaging problem can be computationally expensive and requires use of multiprocessor computers working over many hours to even days to generate single images [16, 22]. While these 3D efforts are useful and necessary to advance the science of microwave imaging, the practical barriers to implementation, including measurement data costs and computation time, have greatly hindered its translation into the clinic and limited them primarily to simulation studies. Alternatively, 2D approaches have proved viable and have been demonstrated in numerous phantom and clinical studies [23–26]. In fact, largely due to continuing computer efficiency advances, 2D techniques are poised to be viable alternatives for conventional modalities in under-resourced settings where cost and portability are significant concerns. In this context, reducing memory requirements and computation time for the 2D algorithm is an important concern.

2D imaging algorithms and system implementations are not new to the microwave imaging community. One notable example is the Semenov group when they were associated with the Carolinas Medical Center in Charlotte, North Carolina. In a relatively early work, they concluded that the reason their imaging technique did not work as well as desired was because of the mismatch between their 2D algorithm and the inherent 3D nature of the actual propagating fields [27]. From that experience, they surmised that they would achieve improved results by transitioning to 3D imaging to avoid the measurement/model mismatch. However, no proof of this being a general conclusion was given. Notwithstanding, significant technological advances have made 2D imaging feasible and attractive. These include: (1) the use of the lossy coupling medium to suppress unwanted multipath signal corruption [28], (2) the use of monopole antennas which can be positioned very close to the target and which we have demonstrated improving the overall images [29], and (3) the use of the log transform which improves the algorithm convergence behavior, eliminates the conversion to local minima, and makes a priori information unnecessary [30, 31]. As such, 2D imaging is poised for an expanded clinical role.

Microwave tomographic imaging algorithms require solving two problems—forward and inverse problems. The forward solver is by far the more computationally expensive part of an iterative image reconstruction algorithm and requires substantial attention to reduce its impact. Additionally, the need to perform many iterations to recover accurate images further motivates the requirement for improving computation time. The most common numerical methods used to solve the forward problem are the finite-difference time-domain (FDTD) [32], finite element method (FEM) [33], and volume integral equations such as the method of moments (MoM) [34]. Recently, the 3D discrete dipole approximation was used as a forward solver for 3D imaging and was found to be highly efficient regarding computational cost and accuracy [35]. We have also introduced the 2D DDA as a forward solver for 2D imaging [36]. As part of that paper, comparisons were performed between the 2D DDA results and those modeled using COMSOL Multiphysics along with comparisons with actual measurements. The agreement was quite good for both. Each numerical method provides important advantages that can be exploited depending on the circumstances. For instance, the finite element method is particularly well suited for situations where nonuniform shaped scatterers are involved. Likewise, the uniform grid formulation of the FDTD problem facilitates fast computation times. While the DDA can be utilized on both uniform and nonuniform grid configurations, as will be demonstrated in this paper, the computational advantages are most significant for the uniform setting. In addition, the associated uniform setting is optimal when the objects in the domain are mostly dielectric in nature. While it would be possible to incorporate metallic scatterers in the domain, for an accurate representation, extra dipoles would need to be deployed on the boundaries of the objects. This would immediately preclude the uniform grid representation. Conveniently, the imaging system developed at Dartmouth [26] essentially provides this feature. The array of monopole antennas is naturally made of metal; however, their radar cross section is sufficiently small that they only slightly perturb the field distribution when another antenna is radiating. In addition, the use of a very lossy coupling medium (glycerin and water mixtures) further dampens any perturbations from scattering off the antennas.

In this work, the two-dimensional discrete dipole approximation (2D-DDA) for calculating the electric field distribution for our microwave imaging system is proposed. The iterative solver for 2D-DDA has the potential to significantly improve the computational speed. A conjugate gradient based method, i.e., the conjugate orthogonal conjugate gradient method (COCG), is used for which the computational cost of the COCG is remarkably reduced when incorporating the fast Fourier transform (FFT). This is made possible because the coefficient matrix for the 2D-DDA is complex, symmetric, and block-Toeplitz after removal of the main diagonal and enables the possibility for employing the FFT after expansion of the block matrices to circulant form. The computation times for the direct and iterative solvers are calculated and have been investigated in this comparison using both MATLAB and C++ implementations. It is useful to compare performance both with an interpretive language such as MATLAB and a classic compiler-based code. While the interpretive code struggles computation time-wise with constructs such as loops, it contains highly optimized matrix operations which can often overcome such disadvantages. These examinations show that the computation time for the 2D-DDA is significantly decreased in the COCG-FFT approach and that the best performance is achieved in C++ using an open source C++ package, FFTW, for fast Fourier transform calculations [37].

## 2. Derivations

In this section, we formulate the 2D-DDA and discuss possible computational efficiency for it as a forward solver of the reconstruction algorithms.

### 2.1. The 2D-DDA for the Forward Problem.

The three-dimensional discrete dipole approximation (3D-DDA) has been widely used for calculation of scattering and absorption properties caused by an external electromagnetic field [38–40]. In the volume integral equations for techniques such as the discrete dipole approximation, an arbitrary geometry Ω is assumed to be a union of small volumes Ω_*i*_ such that $\Omega =\lim_{N\to \infty }{⋃}_{i=1}^{N}\Omega_{i}$ [41]. For the purpose of microwave tomography, the electromagnetic field distribution is expressed in the imaging domain with the forward solver, the discrete dipole approximation. The total electric field at a point *ρ* in the imaging domain is

(1) $$E_{tot}\left(\rho \right)=E_{inc}\left(\rho \right)+E_{scat}\left(\rho \right)$$

where *E_inc_* and *E_scat_* are the incident and scattered electric fields, respectively. The term *E_inc_* represents the electric field propagation due to a waveguide or an antenna for a homogeneous domain. The scattered electric field *E_scat_* is the electric field caused by scatterers in the domain. The Helmholtz equation solution in form of (1) is usually written as [42]

(2) $$E_{tot}\left(\rho \right)=E_{inc}\left(\rho \right)+\int_{\Omega }G\left(\rho ,\rho^{\prime }\right)\left[k^{2}\left(\rho^{\prime }\right)-k_{bk}^{2}\right]E_{tot}\left(\rho^{\prime }\right)d\Omega$$

where *G*(*ρ*,*ρ′*) is the dyadic Green’s function, *k*(*ρ*) is wavenumber at *ρ* ∈ Ω_*i*_, and *k_bk_* is the background wavenumber. The wavenumbers are expressed in terms of the material property and frequency as *k*^2^ = *ω*^2^*μ*_0_*ε*_0_*ε_r_* + *jωμ*_0_*σ*. For the DDA, the idea is to approximate the total electric field, *E_tot_*(*ρ′*) on the right-hand side of (1) such that we assume that the forward model zone consists of multiple number of dipoles. Each dipole represents the macroscopic field *E_tot_*(*ρ′*) at the position of the dipole.

On the macroscopic level, the total electric field is proportional to the polarization *P* via

(3) $$P=\varepsilon_{0}\chi E_{tot}$$

where *ε*_0_ is the permittivity of a vacuum and *χ* = *ε_r_* − 1 is the electric susceptibility. The term $k^{2}(\rho^{\prime })-k_{\mathrm{bk}}^{2}(\rho )$ in Equation (2) is related to *χ* such that

(4) $$k^{2}\left(\rho^{\prime }\right)-k_{bk}^{2}\left(\rho \right)=\omega^{2}\mu_{0}\left(\varepsilon \left(\rho^{\prime }\right)-\varepsilon_{b}\left(\rho \right)\right)\;=\omega^{2}\mu_{0}\varepsilon_{0}\left(\chi \left(\rho^{\prime }\right)-\chi_{b}\left(\rho \right)\right)$$

On the microscopic level, the polarization field is related to dipole moments of individual molecules. Each individual molecule is affected by a local electric field *E_loc_* and gets correspondingly polarized to exhibit dipole moment *p*. The total contribution from all molecules in a unit volume is defined as the polarization P:

(5) $$P=Np=N\varepsilon_{0}\alpha E_{loc}$$

Here *N* is number of molecules per unit volume. The polarizability, *α*, expresses the relationship between the local and macroscopic field. In multiple applications, this interaction has been modeled by the Clausius-Mossotti relation. In the following section, we discuss the polarizability *α* for microwave breast imaging systems.

#### (1) Constant Polarizability (α) for the 2D DDA.

The most common and well-known molecular polarizability, *α*, for the 3D-DDA is the Clausius-Mossotti relationship [43] and is defined for a sphere as

(6) $$\alpha_{CM}=3v\frac{\varepsilon_{r}-1}{\varepsilon_{r}+2}$$

where *ε_r_* and *v* are the relative permittivity and volume of the sphere. The constant term *α* has different formulations depending on the volume and concentration of the medium [41, 44, 45]. Previously, Grzegorczyk et al. [35] used the following 3D Clausius-Mossotti relation for the microwave imaging system at Dartmouth:

(7) $$\alpha_{3D}=3v\frac{\varepsilon_{t}-\varepsilon_{b}}{\varepsilon_{t}+2\varepsilon_{b}}$$

where *ε_b_* and *ε_t_* are the complex permittivities of the background and inclusion, respectively.

**Figure 1:**
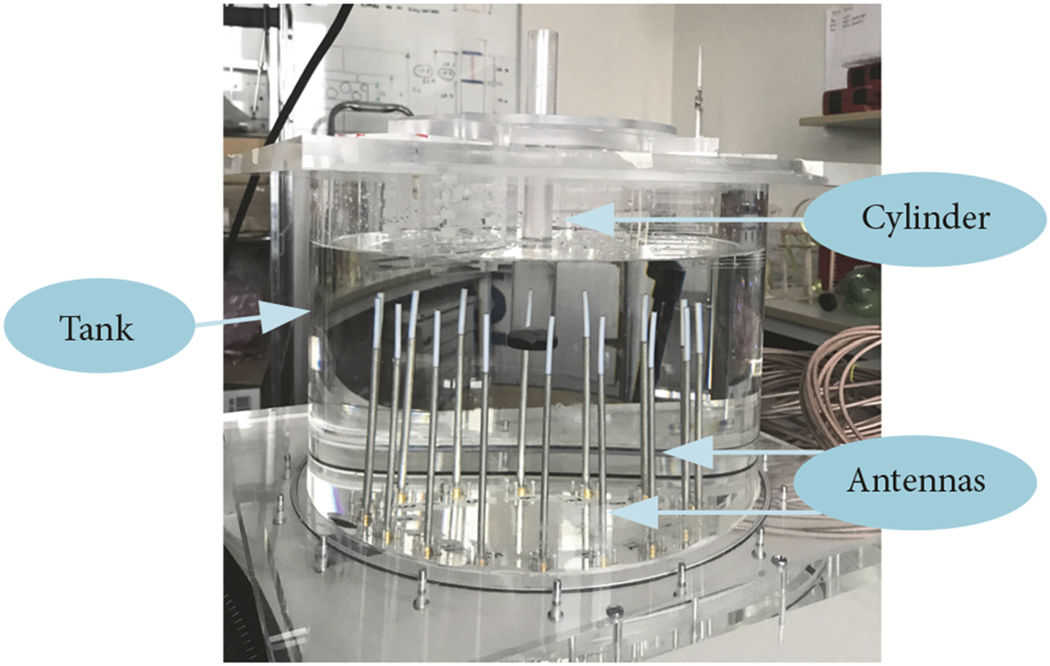
Photograph of the measurement setup including the tank, antennas, and the phantom cylinders.

Since our imaging system shown in Figure 1 consists of a tank filled with a liquid mixture of glycerin and water with different polarization behavior on the molecular level, we select a more general form of the Clausius-Mossotti relationship, the Maxwell-Garnett formula. The main advantage of the Maxwell-Garnett model is that it is also valid for composite media which is the normal situation for many applications including our microwave imaging system. The Maxwell-Garnett formula can be expressed as

(8) $$\frac{\varepsilon_{mix}-\varepsilon_{b}}{\varepsilon_{mix}+2\varepsilon_{b}}=\sum_{k=1}^{K}c_{k}\frac{\varepsilon_{k}-\varepsilon_{b}}{\varepsilon_{k}+2\varepsilon_{b}}$$

In (8), *ε_mix_* denotes the composite medium consisting of *K* media with permittivities, *ε_k_*. The coefficients, *c_k_*, relate the volume of the inclusion to its concentration which is defined as *m/M_r_V* where *V, m,* and *M_r_* are volume, mass, and the molecular weight of the inclusion, respectively. The general formula for the dimensionless *α* in m-dimensions is [46]

(9) $$\alpha =m\frac{\varepsilon_{t}-\varepsilon_{b}}{\varepsilon_{t}+\left(m-1\right)\varepsilon_{b}}$$

Based on (8) and (9), the two-dimensional *α* in the form of the Maxwell-Garnett formula is expressed as

(10) $$\alpha_{2D}≔\frac{\varepsilon_{mix}-\varepsilon_{b}}{\varepsilon_{mix}+\varepsilon_{b}}=\sum_{k=1}^{K}c_{k}\frac{\varepsilon_{k}-\varepsilon_{b}}{\varepsilon_{k}+\varepsilon_{b}}$$

The concentration coefficients *c_k_* are modified based on the area of the inclusion for the 2D case.

#### (2) Incident Electric Field (E_inc_) for the Microwave Breast Imaging.

The microwave imaging system at Chalmers University of Technology has a circular array of monopole antennas acting as both transmitters and receivers (Figure 1). The transmitting antenna is modeled via the electric field distribution caused by an electrical line source (ELS). In this formulation, the 2D ELS is written in the form of

(11) $$E_{inc}\left(\rho \right)=\frac{I_{0}\omega \mu_{0}}{4}H_{0}^{2}\left(k_{b}\left|\rho -\rho_{a}\right|\right)$$

where *I*_0_, *ω*, and *μ*_0_ are the current amplitude, operating frequency, and free-space permeability, respectively. The term |*ρ* − *ρ_a_*| is the distance of a dipole located at position *ρ* from an antenna position *ρ_a_*.

### 2.2. Two-Dimensional Discrete Dipole Approximation as a System of Equations.

The governing equation for the two-dimensional discrete dipole approximation is [36, 39]:

(12) $$E_{\mathrm{tot}}(\rho )=E_{\mathrm{inc}}(\rho )+\sum_{\Omega_{i}}G\left(\rho ,\rho^{\prime }\right)P\left(\rho^{\prime }\right)$$

Inserting (5) into (12) yields

(13) $$E_{inc}\left(\rho \right)=\frac{P\left(\rho \right)}{\alpha \left(\rho \right)}-\sum_{\Omega_{j}}G\left(\rho ,\rho^{\prime }\right)P\left(\rho^{\prime }\right)$$

**Figure 2:**
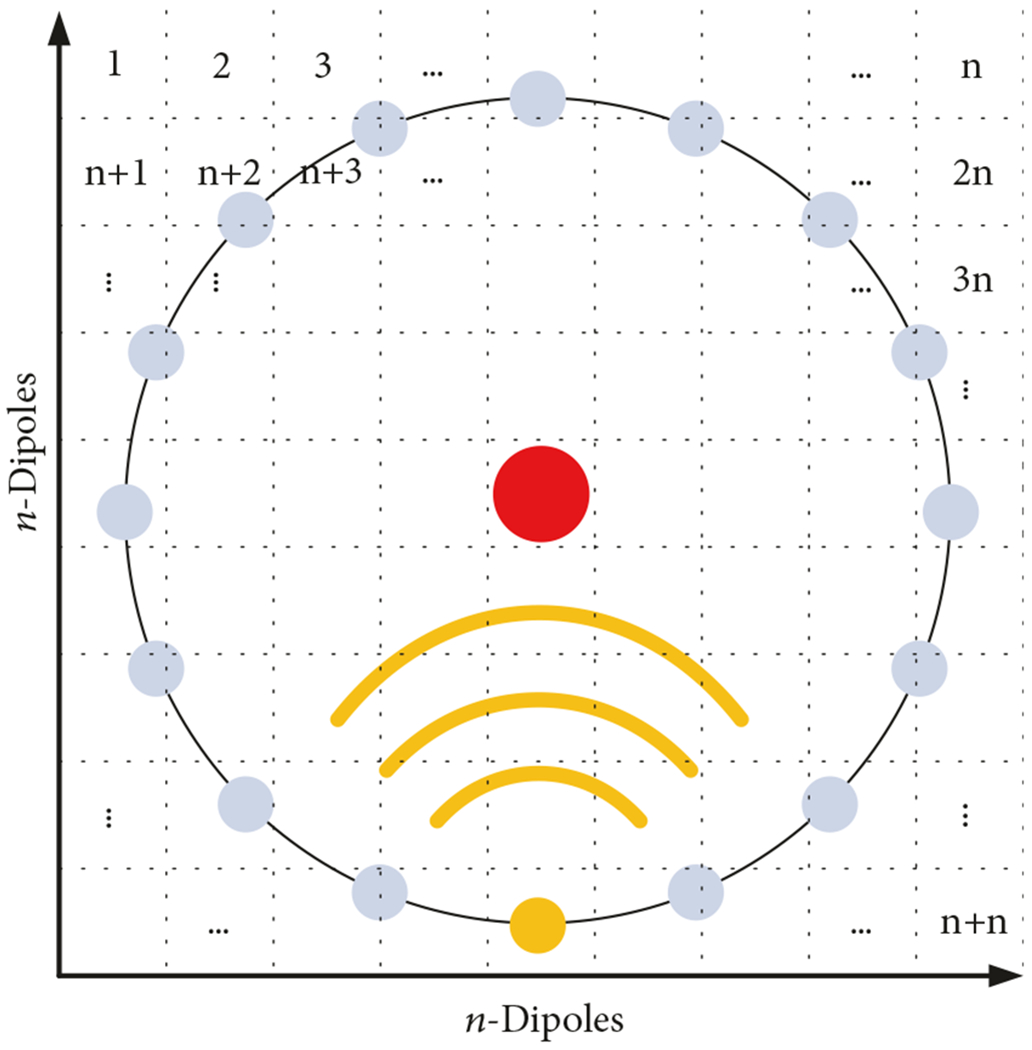
Schematic imaging plane with *n*^2^ equally spaced dipoles on it.

For the 2D-DDA, *G*(*ρ,ρ′*) is the scalar Green’s function for the 2D Helmholtz equation and describes the interaction between two dipoles located at *ρ* and *ρ′*. The relationship is given by

(14) $$G\left(\rho ,\rho^{\prime }\right)=\frac{-j}{4}H_{0}^{2}\left(k_{b}\left|\rho -\rho^{\prime }\right|\right)$$

where $H_{0}^{2}$ is the zero-order Hankel function of the second kind. Discretizing the forward model zone into *N* subdomains, (Figure 2), transforms Equation (13) into its matrix format of

(15) $$\left(\begin{array}{ccccc} \frac{1}{\alpha_{1}} & -G_{12} & -G_{13} & \cdots & -G_{1N}\\ -G_{21} & \frac{1}{\alpha_{2}} & -G_{23} & \cdots & -G_{2N}\\ -G_{31} & -G_{32} & \frac{1}{\alpha_{3}} & \cdots & -G_{3N}\\ \vdots & \vdots & \vdots & \ddots & \vdots \\ -G_{N1} & -G_{N2} & -G_{N3} & \cdots & \frac{1}{\alpha_{N}} \end{array}\right)\left(\begin{array}{c} P_{1}\\ P_{2}\\ P_{3}\\ \vdots \\ P_{N} \end{array}\right)=\left(\begin{array}{c} E_{inc}\left(\rho_{1}\right)\\ E_{inc}\left(\rho_{2}\right)\\ E_{inc}\left(\rho_{3}\right)\\ \vdots \\ E_{inc}\left(\rho_{N}\right) \end{array}\right)$$

One problematic aspect of this formulation is that some terms on the diagonal can approach infinity when any one of the *α*’s goes to zero. This happens when the permittivity at a dipole is exactly the same as that of the background (see Equation (7)). One way to eliminate this problem is to multiply both sides of the equation by *α*. However, this has the unintended consequence of placing the *α* quantities in the off-diagonal terms of the matrix. As will be shown later, we exploit the fact that the off-diagonal terms are only functions of distance and the background permittivity which allows these portions of the matrix to become block Toeplitz and subsequently allows for important optimizations. In fact, for our imaging configuration, we regularly encounter instances where *α* approaches zero. Because of this conflict, we assume a uniform background medium outside of the dipoles where the properties are set to *ε_bk_*. Simultaneously, for our actual measurement system, we utilize a uniform coupling bath everywhere outside the imaging domain, *ε_bath_*. Since our forward model zone is a uniform grid, the properties at all dipoles outside of the imaging domain (a circle in this case) are set to *ε_bath_*. Ideally *ε_bath_* would be set to *ε_bk_* to eliminate all reflections at the grid boundary. However, allowing the *α*’s to go to zero would make the diagonal terms go to infinity. To overcome this, we artificially set *ε_bath_* and *ε_bk_* to be slightly different. This essentially sets many diagonal terms to a large number. In addition, it also implies that there will be nonsignificant signal reflections at the outer boundary of the dipole domain. For the latter challenge, we are fortunate in that our imaging system only uses a highly lossy coupling bath ensuring that waves reflecting back into the DDA and imaging domains will be sufficiently attenuated such that they effectively have no impact on the forward solution. The primary trade-off in this situation is between the level of reflections that can be tolerated and whether the accuracy of the forward solution is adversely affected because the condition number of the matrix in (15) becomes too great. An analysis of this issue is presented in Section 3.1.

### 2.3. Computational Efficient Implementation.

The task of computing the electric field distribution utilizing the DDA involves two primary steps: (a) solving the dipole moments (P) and (b) multiplying P by the matrix in (15) with the diagonal, 1/*α*, terms removed to compute the fields. Because the matrix in (15) is full, the computational order can be as high as 2*N*^3^ /3 when using the standard Gaussian elimination technique [47]. As *N* gets large, this becomes prohibitive and alternatives such as iterative solvers become attractive. In these cases, because the time limiting step involves repetitive multiplication of a length *N* vector by an *N* × *N* matrix, the computational cost is *O*(*N_iter_N*^2^), where *N_iter_* is the number of iterations required for the solution to converge. For this implementation, we exploit the special nature of the matrix derived for the DDA which ultimately allows us to employ the FFT as part of the matrix-vector multiplication to dramatically reduce the computation time within each iteration.

Different iterative solvers have been suggested with most derived from the Krylov subspace methods [39, 48, 49]. Among these methods the conjugate gradient method (CG) is the most popular. In the derivation of the CG algorithm, symmetry and positive definiteness (SPD) are assumed; however, if one of these conditions is not satisfied for the system of equations, the algorithm does not converge to a solution. In our case, the matrix in (15) is symmetric since the off-diagonal terms *G_ij_* are only a function of the background medium and the distances between dipoles *i* and *j*. Unfortunately, the matrix does not meet the requirement of positive definiteness. Alternatively, there are a class of Krylov subspace methods that have been suggested for these conditions [50–57]. For our problem, we have chosen the conjugate orthogonal conjugate gradient method (COCG); it requires one time matrix-vector multiplication per iteration, but similar alternatives such as the conjugate orthogonal conjugate residual (COCR) can also be used with equivalent computational costs [57, 58].

Examination of (15) provides important insights into how best to exploit the COCG method. By breaking the matrix in Equation (15) into its diagonal and off-diagonal components, the left-hand side of (15) can be rewritten as

(16) $$\left(\begin{array}{ccccc} \frac{1}{\alpha_{1}} & 0 & 0 & \cdots & 0\\ 0 & \frac{1}{\alpha_{2}} & 0 & \cdots & 0\\ 0 & 0 & \frac{1}{\alpha_{3}} & \cdots & 0\\ \vdots & \vdots & \vdots & \ddots & \vdots \\ 0 & 0 & 0 & \cdots & \frac{1}{\alpha_{N}} \end{array}\right)\left(\begin{array}{c} P_{1}\\ P_{2}\\ P_{3}\\ \vdots \\ P_{N} \end{array}\right)+\left(\begin{array}{ccccc} 0 & -G_{12} & -G_{13} & \cdots & -G_{1N}\\ -G_{21} & 0 & -G_{23} & \cdots & -G_{2N}\\ -G_{31} & -G_{32} & 0 & \cdots & -G_{3N}\\ \vdots & \vdots & \vdots & \ddots & \vdots \\ -G_{N1} & -G_{N2} & -G_{N3} & \cdots & 0 \end{array}\right)\left(\begin{array}{c} P_{1}\\ P_{2}\\ P_{3}\\ \vdots \\ P_{N} \end{array}\right)$$

**Figure 3:**
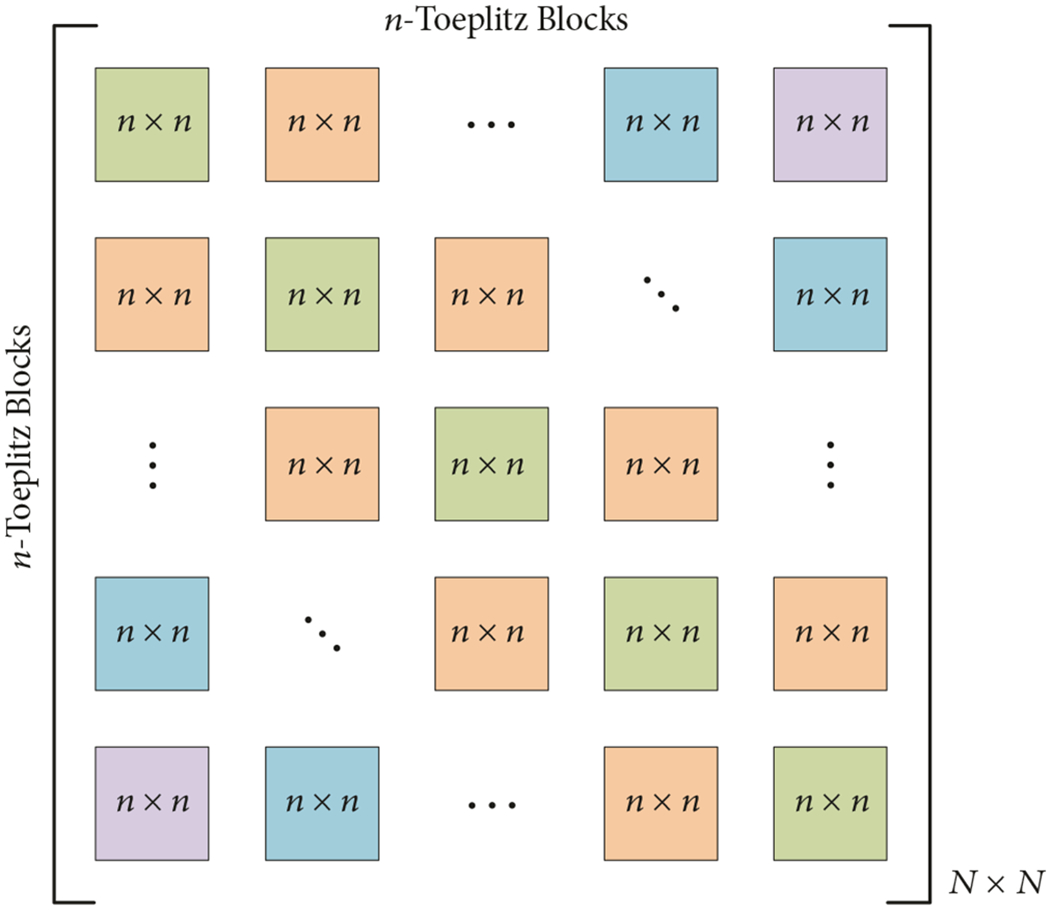
Block-Toeplitz matrix **G**.

The first important observation is that the matrix-vector multiplication in the first term can easily be reduced to an *O*(*N*) vector-vector multiplication. For the right-hand matrix, multiplication times the vector P is normally an *O*(*N*^2^) operation. However, in this situation, the right-hand side matrix, *G*, is block Toeplitz. That is, each of the *N n* × *n* (where $n=\sqrt{N}$) portions of the matrix are themselves Toeplitz matrices (Figure 3). To complete the full matrix-vector multiplication of *G* × *P*, it can be performed by multiplying the smaller Toeplitz matrices times the appropriate portion of *P* and summing the results together afterwards.

Toeplitz matrices are unique in that they are not necessarily symmetric, but they have regular repetition of the individual coefficients, for example:

(17) $$\left(\begin{array}{ccccc} a & b & c & d & e\\ f & a & b & c & d\\ g & f & a & b & c\\ h & g & f & a & b\\ i & h & g & f & a \end{array}\right)$$

**Figure 4:**
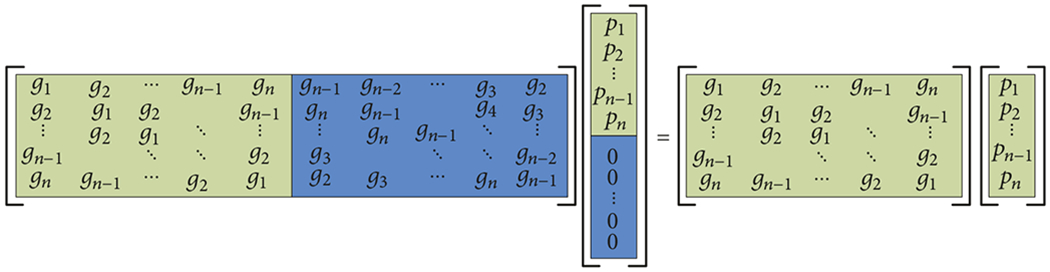
Circulant conversion.

For our case, the Toeplitz matrices are symmetric and are closely related to circulant matrices for which there are highly optimized means for performing matrix-vector multiplications (i.e., the convolution theorem). In this case, the symmetric Toeplitz matrix can be padded both in columns and rows (Figure 4) to produce a circulant matrix:
(18)
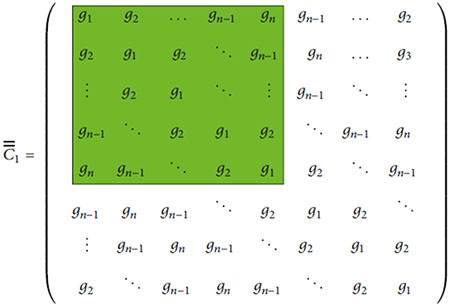

where the upper left-hand side, *n* × *n* matrix was the original block-Toeplitz matrix. By transforming the Toeplitz matrix to this form, several key observations can be made. First, since each row is identical to the next except for a modulo shift, only one row of the matrix needs to be computed and stored. This is a substantial savings in terms of memory requirements. Secondly, because the matrix is now circulant, the convolution theorem can be used to multiply it by the associated portion of the vector (in this case the vector also needs to be padded similarly to the matrix). Taking the inverse FFT of the product of the FFT’s of the first row of the matrix times the associated portion of the vector is equivalent to multiplying the whole matrix times the vector.

## 3. Computational Results

In this section, we discuss two different approaches to optimize the forward solver of the reconstruction algorithms. Additionally, the effects of background medium dielectric properties are investigated and its impact on the electric field distribution is presented.

### 3.1. Study of the Field Distributions as a Function of Permittivity Difference.

As mentioned in Section 2.2, one challenge in using Equation (15) is the treatment of the diagonal of the system of equations coefficient matrix. As the *α* terms tend to zero, implying that the electrical properties of background liquid are the same as those of the dipoles, the corresponding diagonal terms go to infinity. For medical microwave imaging systems, a coupling liquid reducing the contrast between skin tissues and its surrounding is used. Utilizing a coupling liquid that has identical dielectric properties to that of the outside results in a matrix with infinity values on its diagonal. To accommodate this challenge, we set the properties of the dipoles within the grid to something slightly different than that for the background. This problem motivates us to study the range of acceptable permittivity values for its surrounding. For this analysis, the permittivities of the background and coupling medium are denoted as *ε_bk_* and *ε_bath_*, respectively.

**Figure 5:**
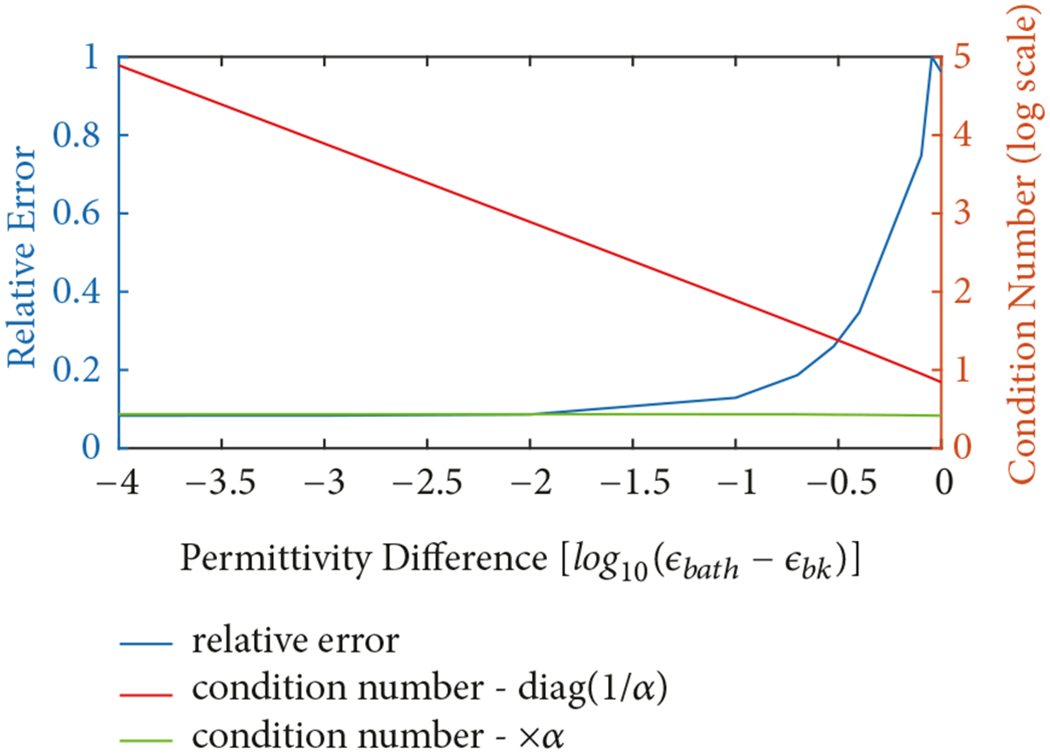
The relative error of the total solution, condition number of the matrix with 1/*α* on the diagonal, and the condition number of the matrix that has been multiplied by *α* all as a function of the logarithmic difference in permittivity between the bath, *ε_bath_* = 22 and the background. For the permittivity difference scale, −4 corresponds to a background permittivity of 21.9999 and a value of 0 corresponds to a background permittivity of 21, respectively.

**Figure 6:**
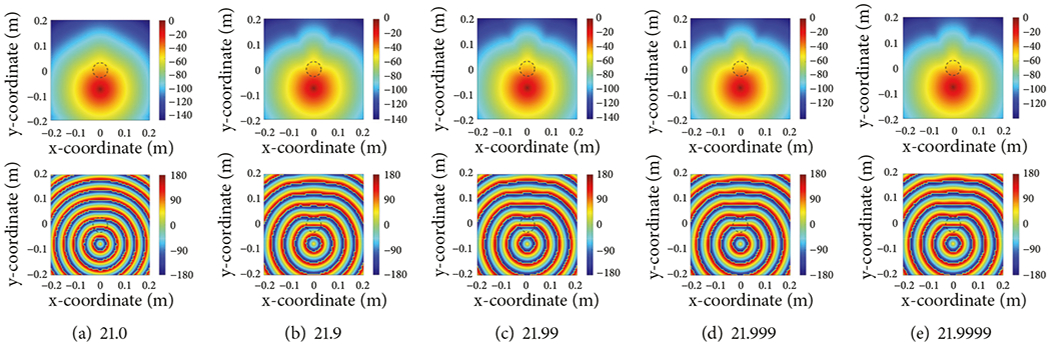
Plots of magnitude (top) and phase (bottom) distributions for *f* =1.3 GHz, *σ* = 1, *ε_r,t_* = 40, *d* = 6 (cm), forward zone of size of 40 cm and #6561 number of dipoles, for multiple background permittivity values, *ε_r,bk_*, in range of 21 to 21.9999. The inclusion is located at (0m, 0m) and its outline is indicated by the dashed lines.

In this situation, we set the permittivity values for the grid region directly surrounding the imaging domain to *ε_bath_* and that for the surrounding background to a value slightly lower than that. We compute the electric field distributions for a single, large inclusion (*ε_inc_* = 40, and *σ* = 1.0 S/m) and the bath properties of *ε_bath_* = 22 and *σ*= 1.0 S/m. The imaging domain consists of 6561 dipoles. The antenna array is located on a circle of diameter 15.2 cm and the target is positioned at the origin. In this situation, the background permittivity is varied from 21.9999 down to 21 where the difference between *ε_bath_* and *ε_bk_* is increased in increments of multiples 10 (i.e., *ε_bk_*= 21.9999, 21.999, 21.99, 21.9, and 21.0) and the conductivity is kept constant at *σ*= 1.0 S/m. Figure 5 shows plots of (a) the condition number of the matrix using the formulation in (15), (b) the condition number of the matrix using the formulation where (15) has been multiplied by *α*, and (c) the relative error for the first formulation, all as a function of the difference in permittivity between the bath and background (log scale). The condition number is a useful metric because it provides a good measure of the digital accuracy of the inversion process utilizing a particular matrix [47]. Note that the condition number of the case where the matrix is multiplied by *α* does not change appreciably since the values on the diagonal remain within a relatively tight bound. The field distributions (both amplitude and phase) for the cases of background permittivities of 21, 21.9, 21.99, 21.999, and 21.999 are shown in Figures 6(a)–6(e). For the most part, these distributions are quite similar. Except for *ε_bk_* = 21, the amplitude plots all show a primary lobe towards 12:00 (clock-face orientation) with slight nulls to either side of it. The *ε_bk_* = 21 case does exhibit a slight bulge towards 12:00, but the nulls have been smoothed over. The phase distributions show roughly circular patterns with the sharp changes from red to blue designating phase wrapping as the distributions jump from −180° to +180°. These distributions are roughly circular, with the cases for *ε_bk_* ≥ 21.9 exhibiting a slight flattening in the 12:00 direction. The *ε_bk_* = 21 distribution is more circular for the entire region. From the visual examination in Figures 6(a)–6(e) and the error and condition number plots in Figure 5, it would appear that background permittivity values of 21.99 or greater would be suitable. However, our preference is to keep the condition number to a modest level, so we choose a value of 21.99 to restrain the condition number while simultaneously keeping the error low.

### 3.2. Computational Efficiency with the FFT.

We have implemented the 2D-DDA using both MATLAB and C++. The iterative and direct solutions for the given system of equations have also been calculated. Since the main concern on the choice of these algorithms stems from optimizing computation time to obtain vector *P*, we have calculated and compared the computation times for each implementation and method with different numbers of dipoles. The computation times are divided into three categories based on the complexities *N*^3^, *N*^2^, and *N* log (*N*) for the direct solvers, the COCG iterative algorithm and the FFT-COCG algorithm, respectively. The computation time for the COCG-FFT implemented in C++ is processed in two separate software packages, Armadillo and FFTW. Armadillo is an open source C++ library for linear algebra and scientific computing which provides a high level syntax and is balanced between speed and user friendliness [59, 60]. FFTW is a C subroutine library for computing the discrete Fourier transform (DFT) in one or more dimensions, of arbitrary input size, and of both real and complex data [37]. These two packages have been used in our implementation in this study. Table 1 shows the computation times for different solvers in MATLAB and C++. For these simulations, the forward model zone contains *N* number of dipoles, where *N* = 441,1681, and 6561. The computation times for these cases are given in Table 1. Table 1 shows that the MATLAB direct solver is more efficient compared to that of the C++ implementation using the Armadillo package. Generally, for the two smaller matrices, MATLAB is more efficient than C++. However, when using larger matrices with sufficient numbers of dipoles in the imaging domain (*N* = 6561) to ensure accuracy, the computation times for the direct and iterative solvers in MATLAB show that the time savings are on the order of a factor of two. Using the FFT-COCG in MATLAB, the time decreases by a factor of three. Our observations show that there is a significant difference in computation time between the Armadillo and FFTW packages using the built-in FFT algorithm.

### 3.3. two orders of magnitude faster than a conFurther Optimizations for Our Specific Microwave Imaging Problem.

In this section, the optimal number of dipoles utilized in the imaging domain is investigated. In the previous section, it was convenient to sequentially double the number of dipoles to more easily identify the optimal number of dipoles. However, there may be more convenient intermediate values which have improved efficiency, especially with respect to the FFT.

For the block-Toeplitz matrices in the case with 6561 dipoles, the square Toeplitz matrices are 81 × 81. To convert these matrices to circulant ones, the size of the matrix needs to be nearly doubled in width and height along with duplication of coefficients to that in (18). In this case, the conversion means adding 79 columns with appropriate coefficients, before performing the actual FFT. In this form, the number of columns and the length of each column increases to 160.

**Figure 7:**
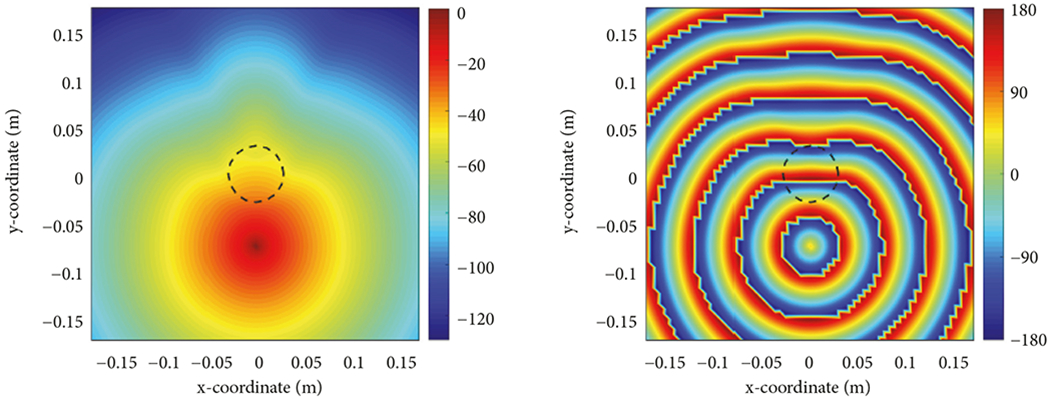
Plots of magnitude (left) and phase (right) distributions for *f* =1.3 GHz, *σ* = 1, *ε_r,t_* = 40, *d* = 6 (cm), for forward zone of size 35.2 × 35.2 cm and 4225 dipoles, for background permittivity value of *ε_r,bk_* = 21.99.

The fast Fourier transformations of the first row of the matrix and the column vector also require zero-padding procedures to the next power of 2 to allow use of the FFT. In this way, a vector with the size of 160 would be converted to the one of size 256. One way to optimize these cases is to restrict our number of dipoles in a way that does not require significant zero-padding. One example of exploiting this observation is to reduce the overall grid size to 65 × 65 dipoles. Without reducing the physical size of the grid, this would imply a node-to-node spacing of 6.3 mm instead of the previous 5.0 mm spacing, an increase of 25%. However, by decreasing the grid size by only 12% (corresponding to spacings of 5.5mm and an overall grid size of 35.2cm x 35.2cm), the overall change to the accuracy is only minor, and we accomplish the goal of improved efficiency. Figure 7 shows the magnitude and phase distributions for the same case in Figure 6(c), the background permittivity of 21.99, except that the overall grid size is 35.2 (cm) × 35.2 (cm). While the physical boundary is closer to the antennas, the artifacts from the boundary still have minimal impact on the fields inside the array of antennas. In terms of efficiency, Table 2 shows the performance times for this grid size. For all solution techniques, this size has substantial time improvement over the previous 6561 case, especially when the FFT is exploited, by as mush as a factor of 3*x* depending on method. This is a substantial improvement.

Moreover, for the iterative algorithms such as COCG, the number of iterations is not independent of prescribed accuracy tolerance levels. To reduce the computation time, we studied the effects of the error tolerance level on the computation time as well as the overall accuracy. Table 3 shows the corresponding computation times for tolerances of 1e-5 and 1e-3 for the 6561 dipole grid. For all implementations, there are significant time improvements, with the C++ times utilizing the FFT algorithm improving on the order of 35%.

## 4. Conclusion

We have implemented a version of the DDA for computing the forward solutions for a configuration used in our current 2D tomographic imaging system. 2D is intriguing in that it has already been successfully implemented for phantom and animal experiments along with considerable clinical studies. Even without the speed enhancements from the techniques presented here, the 2D approach is already considerably faster than any existing 3D inverse technique which positions it well with regard to being the foundation for a low cost and portable system that would be suitable for under-resourced settings.

We have previously demonstrated that the DDA is accurate for forward solution computation for our imaging algorithm. This study builds on that experience and formulates the problem in ways that facilitate dramatic speed optimization. Chief among these enhancements is the notion of utilizing conjugate gradient based iterative solvers in conjunction with breaking the core matrix into a simple diagonal matrix and a separate one that can be further broken into block-Toeplitz matrices. Through standard techniques, these smaller matrices can be easily expanded into circulant matrices for which the FFT can be employed to speed up the matrix/vector multiplications utilizing the convolution theorem.

Further enhancements including judicious selection of the grid size and analysis of appropriate error tolerance levels for the conjugate gradient type iterative process allow the forward solution time to decrease to the order of 0.1 second which is almost two orders of magnitude faster than a conventional COMSOL approach that is considered to be efficient. These computation time improvements along with significant reductions in memory usage because of the nature of circulant matrices make this an attractive approach for simplifying and greatly speeding up the image reconstruction process.

## Figures and Tables

**Table 1:** Computation times (s).

	Direct		COCG		COCR		COCG-FFT			COCR-FFT		
#dipoles	MATLAB	C++ (Armadillo)	MATLAB	C++ (Armadillo)	MATLAB	C++ (Armadillo)	MATLAB	C++ Armadillo	FFTW	MATLAB	C++ Armadillo	FFTW
441	0.0045	0.0540	0.0029	0.0033	0.0034	0.0037	0.0413	0.0029	0.0069	0.04123	0.0030	0.0069
1681	0.1790	2.1660	0.0790	0.0632	0.0830	0.0680	0.1869	0.0565	0.0600	0.1851	0.0589	0.0549
6561	5.3340	57.8030	1.5296	1.4693	1.6020	1.5353	1.4603	1.2336	0.6431	1.4410	1.3612	0.6512

**Table 2:** Computation times for the power of 2 optimized grid size.

	Direct		COCG		COCR		COCG-FFT			COCR-FFT		
#dipoles	MATLAB	C++ (Armadillo)	MATLAB	C++ (Armadillo)	MATLAB	C++ (Armadillo)	MATLAB	C++ (Armadillo)	FFTW	MATLAB	C++ (Armadillo)	FFTW
4225	1.7129	31.7440	0.6444	0.5555	0.6461	0.5836	0.7637	0.5689	0.2746	0.5785	0.5366	0.2758

**Table 3:** Comparison of computation times as a function of conjugate gradient scheme accuracy tolerances.

	Tolerance = 1e-5				Tolerance = 1e-3			
	COCG	COCG-FFT	COCR	COCR-FFT	COCG	COCG-FFT	COCR	COCR-FFT
MATLAB	0.3351	0.4090	0.3749	0.4042	0.2061	0.2694	0.2344	0.2613
C++	0.3470	0.1655	0.3741	0.1665	0.3237	0.1073	0.2648	0.1080
